# Treating Apnea of Prematurity

**DOI:** 10.7759/cureus.21783

**Published:** 2022-01-31

**Authors:** Joseph Pergolizzi, Alexander Kraus, Peter Magnusson, Frank Breve, Kailyn Mitchell, Robert Raffa, Jo Ann K LeQuang, Giustino Varrassi

**Affiliations:** 1 Cardiology, Native Cardio Inc., Naples, USA; 2 Research and Development, Enalare Therapeutics, Inc., Lorrach, DEU; 3 Cardiology, Center of Research and Development Region Gävleborg/Uppsala University, Gävle, SWE; 4 Medicine, Cardiology Research Unit, Karolinska Institutet, Stockholm, SWE; 5 Pharmacy, Temple University, Philadelphia, USA; 6 NEMA Research Inc., Naples, USA; 7 School of Pharmacy, Temple University (Emeritus), Philadelphia, USA; 8 NEMA Research, Inc., Naples, USA; 9 President, Paolo Procacci Foundation, Rome, ITA

**Keywords:** doxapram, neonatal intensive care, neonatology, caffeine, preterm birth, apnea of prematurity

## Abstract

Premature babies often suffer apnea of prematurity as a physiological consequence of an immature respiratory system. Hypercapnia may develop, and neonates with apnea of prematurity are at an increased risk of morbidity and mortality. The long-term effects of apnea of prematurity or their treatments are less clear. While a number of treatment options exist for apnea of prematurity, there is no clear-cut “first-line” approach or gold standard of care. Effective treatments, such as caffeine citrate, carbon dioxide inhalation, nasal continuous positive airway pressure, nasal intermittent positive pressure ventilation, and others, may be associated with safety concerns. More conservative treatments are available, such as kangaroo care, postural changes, and sensory stimulation, but they may not be effective. While apnea of prematurity resolves spontaneously as the respiratory system matures, it can complicate neonatal care and may have both short-term and long-term consequences. The role, if any, that apnea of prematurity may play in mortality of preterm neonates is not clear.

## Introduction and background

Approximately 11% of births globally are preterm, which s defined as birth before 37 gestational weeks [1]. Premature birth does not accelerate respiratory development, meaning that these neonates must rely on fetal pulmonary mechanisms, which often leads to a form of respiratory instability known as apnea of prematurity (AOP). Increasingly, preterm infants of lower gestational age survive, making AOP more prevalent in the neonatal intensive care unit (NICU). A variety of treatments may help stimulate respiration, but great care is warranted as the immature respiratory systems of preterm infants can easily be injured by aggressive approaches. AOP is a potentially life-threatening sign associated with preterm birth, which resolves spontaneously as the respiratory system matures [1]. AOP may be more complex and more dangerous than is commonly considered. Complications associated with AOP may involve the physiological consequences such as altered neonatal ventilatory responses to hypoxia and hypercapnia, and disruptions to neonatal sleep architecture [2].

AOP has been defined as the abrupt cessation of breathing for at least 20 seconds, accompanied by both bradycardia and oxygen desaturation in an infant with a gestational age < 37 weeks (Table 1) [3]. It may be accompanied by a clinically significant cardiopulmonary event (CSCPE), defined as 20 seconds without respiration or 10 seconds without respiration accompanied by a heart rate under 80 beats per minute or an oxygen desaturation level <85% [4]. The incidence of AOP and CSCPE increases with lower birth weight, affecting 25% of neonates weighing less than 2,500 g at birth [5]. Apnea may be central, obstructive (blocked airways), or mixed; central and mixed apnea are more frequently observed. However, clear distinctions between these three broad categories may sometimes become blurred [6].

**Table 1:** The incidence of AOP is inversely related to gestational age and birth weight [5]. AOP, apnea of prematurity

Gestational age	Incidence of apnea of prematurity	Birth weight
34-35 weeks	7%	
32-33 weeks	15%	
30-31 weeks	54%	
<29 weeks	~100%	<1,000 g

The fetus lives in an environment with very low oxygen (PaO2 of 23-27 mmHg) and transitions at birth abruptly into an oxygen-rich environment, which necessitates a rapid and radical change in ventilation. Neonatal oxygen therapy must provide adequate oxygen for tissue perfusion without causing oxygen toxicity and retinopathy of prematurity [7]. Babies in distress administered 100% oxygen at delivery may experience oxidative stress as well as toxicity involving the eyes, the brain, or the lungs [7,8]. Preterm infants may lack chemoreceptor sensitivity, respiratory control system, and adequate synaptic connections in order to make this ventilatory adjustment safely and quickly [2]. Moreover, preterm infants may develop hypercapnia, which may prolong expiration without increasing the respiratory rate. In fact, poor hypercapnic response is more common in premature infants with AOP than in those without AOP [2]. AOP appears to be more complex than a simple breathing dysfunction related to an immature anatomy.

If left untreated, AOP can lead to failure to thrive, developmental delays, and other forms of morbidity and mortality. It increases the neonate’s likelihood of having other disorders such as respiratory failure, pulmonary hemorrhage, cardiovascular problems, intracranial hemorrhage, and sudden death [9]. While AOP typically resolves as the infant matures, in some cases, apneic events may persist beyond infancy; in a cross-sectional study of 137 children between the ages of 4 and 6 years, obstructive sleep apnea was significantly more common in preterm than full-term patients [10]. The association between AOP and neurodevelopmental delays is difficult to establish because preterm infants may have multiple underlying health problems and developmental challenges separate from AOP. In most cases, AOP resolves by 36 weeks of postmenstrual age, but this is not a universally observed outcome [11]. Indeed, there is a paucity of data about the natural course of AOP, although the condition is far from rare. The purpose of this narrative review is to summarize and contextualize the current treatment options for AOP.

## Review

A literature search was conducted using PubMed for the keywords “apnea of prematurity” with results delimited to randomized clinical trials (n=46 results). The Cochrane Library database was searched for meta-analyses of data by searching for “apnea of prematurity” in the title, abstract, or keywords (n=37). Google Scholar was searched for “apnea of prematurity” and “apnea of prematurity guidelines.” The last searches of these databases were performed in June 2021. Embase was searched on July 22, 2021, for “apnea of prematurity” from 2000 to 2021, with results limited to controlled clinical trials and randomized controlled trials (n=56). Results were excluded if they were not in English; if articles did not report randomized trials, clinical trials, or meta-analyses; or if the authors did not specifically address treatment modalities for AOP. The emphasis of our search was on treatment options. Bibliographies of articles were also searched. There was some duplication in results. In total, 96 articles were used. Since AOP increases a neonate’s probability of morbidity and mortality [9], it is important to find safe and effective ways to treat this condition. The natural history of AOP is such that it often resolves on its own, but the most beneficial course of action in this setting is not clear. The main currently available treatments are described in the next sections.

Methylxanthine

The only treatment for AOP approved by the U.S. Food and Drug Administration (FDA) is caffeine citrate. Caffeine is one of several methylxanthine drugs used in this setting, similar to theophylline and aminophylline [12]. Among these agents, caffeine has a higher therapeutic ratio, good enteral absorption, a longer half-life, and fewer adverse effects; its efficacy and tolerability make it the first choice among methylxanthines [13]. Since 2013, caffeine has been used more often than other drugs to treat AOP [14]. As a central nervous system stimulant, methylxanthines cross the blood-brain barrier and act as antagonists at the adenosine receptors [15]. Genetic differences may make some neonates more or less sensitive to these agents, accounting for an observed variability in response [16]. It has been speculated that methylxanthines administered to a neonate may have adverse effects on the cardiovascular and central nervous systems [17]. An international study of 13 academic hospitals followed the progress of 870 neonates with AOP who were administered caffeine citrate for the next 11 years; the study used regression models to ascertain the potential neurobehavioral effects of neonatal administration of caffeine [18]. Overall, neurobehavioral results were similar in the caffeine and control groups, but the caffeine group had better performance in fine motor coordination, visual-motor integration, and visual-spatial organization than the controls. Attention, intelligence, and general behavior were similar between groups [18]. In another study, caffeine therapy did not significantly affect academic performance, motor skills, or behavioral impairments up to middle-school age compared to placebo patients in a study of 920 neonates with AOP followed for 11 years [19].

Caffeine is labeled for short-term use for a limited range of gestational ages, but there is limited specific guidance regarding when to start treatment, dose and duration of treatment, when treatment should be discontinued, and whether caffeine might have adverse effects [3]. The long-term effects of caffeine administered to the developing brain are not fully known, and it has been suggested that the positive respiratory benefits of caffeine in preterm neonates may be outweighed by caffeine’s adverse effects on the central nervous system [20]. In a retrospective cohort study of 109 infants with a gestational age of <31 weeks and birth weight under 1,500 g, the administration and duration of caffeine citrate therapy were associated with osteopenia of prematurity, a potentially serious condition [21]. Dosing can be particularly challenging because the preterm population has limited hepatic, renal, and respiratory function. A meta-analysis (n=1,515 patients in 13 randomized clinical trials) found that higher doses (10-20 mg/kg daily) were associated with greater efficacy than lower doses (5-10 mg/kg daily) and that higher doses were more closely associated with withdrawal from the ventilator, but higher doses were also more associated with tachycardia than lower doses [22]. In another study of caffeine administered to 120 preterm infants (<32 weeks gestational age), high-dose caffeine therapy (loading dose of 40 mg/kg and then 20 mg/kg daily) was associated with a significant decrease in extubation failure, apnea rates, and days of documented apnea compared to low-dose caffeine (loading dose 20 mg/kg and then 10 mg/kg daily) [23]. A clinical study compared the same doses of caffeine to treat AOP in 78 preterm infants in the NICU [24]. There was no significant difference in the frequency or total days of apnea between groups, and adverse events were similar as well [24]. While high doses were not associated with worse outcomes, this study suggests that high-dose caffeine therapy may not confer any benefits in this population. By contrast, a study of 111 preterm infants with AOP randomly assigned to high-dose (20 mg/day) or low-dose caffeine therapy (10 mg/day) found that the high-dose group had significantly lower rates with respect to extubating failure (16.7% vs. 36.8%), age at extubating (8.2±2.1 days vs. 10.7±2.3 days), duration of invasive ventilation, duration of ventilation prior to extubating, and number of days of apnea (1.8±1.3 vs. 3.2±1.1 days), with a similar rate of adverse events [25]. In contrast to other studies, this study suggests that a higher maintenance dose of caffeine is associated with better outcomes.

The largest study of caffeine in preterm infants to date, the Caffeine for Apnea of Prematurity (CAP) Trial, randomized 2,006 neonates with birth weights ranging from 500 to 1,250 g to the caffeine group or placebo for the first 10 days of life. Caffeine was administered as a loading dose of 20 mg/kg followed by a maintenance dose of 5 mg/kg/day, which could be increased to 10 mg/kg/day in cases of persistent apnea [26]. The caffeine group had a shorter duration of mechanical ventilation, lower rate of bronchopulmonary dysplasia (BPD), and improved neurodevelopmental outcomes at 18 months; these advantages were less pronounced at five years, but benefit for the caffeine group was still shown [27].

In a randomized, single-center, controlled study of AOP prophylaxis, 26 preterm infants received either 20 mg/kg caffeine citrate as a loading dose followed by 5 mg/kg daily over 10 days or were in a control group [28]. AOP occurred significantly less frequently in the caffeine compared to the control group, with only 15% of the caffeine group (n=4) developing AOP versus 62% (n=16) in the control group [28]. A randomized study of 90 preterm infants evaluated whether prophylactic caffeine treatment, defined as caffeine administered in the first 72 hours of life, conferred benefits over therapeutic caffeine administered only to infants with apnea and/or who required mechanical ventilation. The prophylactic caffeine group had a shorter duration of oxygen therapy, shorter duration of invasive or noninvasive ventilation, a lower incidence of moderate BPD, and a shorter length of hospital stay [29].

Moreover, caffeine was associated with a significantly reduced risk of motor impairment versus placebo (p=0.009) in neonates with AOP [19]. The composite endpoint of death or disability at five years was statistically similar in 1,640 children randomized to be treated with caffeine or placebo for AOP [27]. The rate of cognitive impairment was less at five years than 18 months in both groups and not different between groups [27]. In the children in the CAP study (n=1433), the rate of developmental coordination disorder was evaluated in children who had received caffeine (n=735) or placebo (n=698) five years later. The rate of developmental coordination disorder was significantly lower in the caffeine group than the placebo group (11.3% vs. 15.2%, p=0.032) [30]. In a study of seventy 11-year-old children who had been born preterm (≤1,250 g weight at birth) and were treated with caffeine or placebo for AOP, imaging evidence showed that caffeine-treated children had a smaller corpus callosum than placebo patients, although volumetric development of the brain was similar between groups [31]. The clinical significance of this finding is not known, nor is it known if this difference persists beyond age 11. In another study, 201 children who had been treated as preterm neonates with caffeine citrate or placebo were evaluated at ages between 5 and 12 years for abnormalities in sleep architecture or breathing during sleep [32]. It was found that caffeine had no long-term effects on sleep duration or sleep apnea, although all of the preterm infants in this study had similar and elevated risks for obstructive sleep apnea and periodic limb movements during sleep at older age [32].

Caffeine has interindividual pharmacodynamic variations in preterm neonates, with the plasma elimination half-life estimated to be about 100 hours [13]. There is no expert consensus as to when caffeine treatment should be started in preterm neonates, but it is often initiated in the first few hours or days of life [33]. Early treatment with caffeine is associated with a significantly decreased need for invasive ventilation or duration of mechanical ventilation, but early administration may pose risks for the developing brain and central nervous system [33]. Likewise, there is no clear expert consensus as to when caffeine treatment should be discontinued. In a study of neonates of 26 to 32 weeks’ gestational age with AOP, 120 infants were randomized to be treated with caffeine until the seventh apnea-free day or for a fixed time period that culminated at 34 weeks of postmenstrual age [34]. Results were similar between groups, and the study suggests that caffeine may be stopped as early as 33 to 34 weeks of postmenstrual age without risking recurrence of apnea [34]. A survey of caffeine use in neonates with AOP in the United States found that caffeine was most frequently discontinued at the postmenstrual age of 34 weeks (62%) and that most of them were not discharged home on caffeine therapy (70%) [35].

The dose of caffeine and the toxicity threshold are not well defined [13]. Issues with caffeine citrate may involve dose calculation errors, as the product is usually available only in a few commercially available sizes and neonatal doses are usually extemporaneously compounded. It is also important to recognize that caffeine citrate is only available in a preservative-free formulation, and therefore left-over compounded product must be promptly discarded. Elimination of caffeine increases nonlinearly after birth of a preterm infant up to the age of around six weeks [36]. Peak plasma concentrations occur in within one hour, and caffeine, being hydrophobic, tends to distribute rapidly in the body and not accumulate in tissue. Preterm neonates metabolize caffeine mainly by N-demethylation, and females tend to metabolize caffeine more rapidly than males [13]. At up to 38 weeks’ gestational age, the hepatic enzymes are immature and can reduce caffeine metabolism, prolonging the plasma half-life of caffeine. Hepatic enzymes do not function at an adult level until the age of approximately four months [37].

Aminophylline is a bronchodilator that helps relax airway muscles. In a retrospective study of 206 preterm infants treated with aminophylline for AOP, it was found that 62% received effective therapy and 26% had adverse events. Higher rates of adverse events could be associated with low birth weight and high serum concentrations of the drug [38]. The effects of aminophylline on cardiac parameters in neonates appear to be similar to those of caffeine [39]. In a randomized, double-blind study, 87 preterm neonates with AOP (gestational age of 27-32 weeks) were treated with either theophylline plus 0.5 L/m room air via nasal prongs or placebo with 0.5 L/min room air with carbon dioxide (about 1% inhaled) for three days [40].

Blood transfusion

Red blood cell transfusions have been reported as a treatment of AOP, but there is little evidence to support their effectiveness [41]. In fact, since neonates are already hyperglobulinemic, this treatment seems inappropriate. An infusion of red blood cells is thought to treat AOP by boosting the oxygen content of the blood, and, in that way, enhancing oxygenation of the tissues [42]. Unblinded studies have produced conflicting results [43,44]. Furthermore, blood transfusions delivered to low birth-weight neonates for any reason are associated with an increased risk of BPD and necrotizing enterocolitis [44].

Carbon dioxide inhalation

Since apnea occurs when the baseline level of carbon dioxide falls below the apnea threshold, inhalation of carbon dioxide is sometimes used in mammals as a way to physiologically induce breathing. In a study of 42 preterm infants, inhaled carbon dioxide 0.8% was found to be as effective as theophylline in terms of reducing apneic episodes without altering cerebral blood flow velocity [45]. However, a randomized double-blind study of 87 preterm infants with AOP found inhaled carbon dioxide therapy ineffective [40]. Neonates may accommodate themselves to carbon dioxide treatments, making them less effective over time, and the longer-term consequences of this type of treatment have not been evaluated.

Creatinine supplementation

Preterm infants with AOP were randomized to be treated with creatinine supplementation or placebo (n=38). Creatinine supplementation was well tolerated but had no significant effect on bradycardia incidence or oxygen desaturation [46].

Device-based treatment (neuromodulation)

It is known that limb movements can drive respiration; for example, walking stimulates breathing in humans [47]. Since this effect occurs with passive limb movement as well, gentle vibrations were used to stimulate the proprioceptors on the palms of the hands and soles of the feet of preterm infants. The concept was that vibrations would activate a proprioceptive fiber discharge similar to such that might occur with ambulation, involving a reflexive coupling of respiration and movement. In a study of 19 preterm infants (≥23-34 weeks’ gestational age), this treatment was shown to reduce AOP, reduce oxygen desaturation, and result in fewer instances of bradycardia. The neuromodulatory approach is noninvasive and was well tolerated [47].

Doxapram

Doxapram, a respiratory stimulant, is sometimes administered to preterm infants with AOP at doses of about 1.5 mg/kg per hour [48], but doxapram has dose-dependent side effects that must be carefully considered [49]. A systematic review of doxapram to treat AOP in neonates of <34 weeks’ gestational age (n=28 studies, 1,994 patients) found that doxapram conferred a benefit in terms of apnea rate but with the risk of dose-related adverse events [50]. These investigators could reach no conclusions on the safety or efficacy of this drug in this population and therefore did not recommend its routine use [50].

In preterm infants, doxapram is considered an appropriate but third-line treatment for AOP, suitable for use if the neonate does not respond to caffeine or continuous positive airway pressure (CPAP) [51]. In a randomized double-blind study of 85 preterm infants with AOP for whom neither caffeine nor CPAP was effective, neonates were treated with doxapram and followed for four days. The study found that regulating doxapram dosing by considering infant weight and sex did not significantly increase plasma levels of the drug in the therapeutic range but did improve efficacy with 76% of these infants responding to doxapram therapy compared to 56% of controls (p<0.001) with no adverse events observed [51]. In a study of 11 preterm infants randomized to intravenous doxapram or placebo, the doxapram group had fewer treatment failures within 48 hours, but this study only evaluated short-term responses [52]. In a study of 1,501 neonates of <32 weeks’ gestational age who were treated with doxapram over the course of five years at a single center in the Netherlands, the majority of those (64.8%) treated with doxapram did not need intubation. No neonates in this study died [53].

Kangaroo care

Kangaroo care, the skin-to-skin contact between parent and newborn infant, has gained acceptance as a way to improve the infant’s vital signs and clinical status, particularly for healthy babies. Its role in the treatment of AOP is not clear, as the results of studies of kangaroo care for reducing AOP have been mixed [54-56]. In fact, kangaroo care may interfere with adequate respiratory monitoring of preterm infants with AOP unless special steps are taken [57]. However, kangaroo care may reduce morbidity and mortality in low birth weight infants [58] and promote weight gain [59].

Less-invasive surfactant administration

Pulmonary surfactants are substances that prevent the pulmonary air sacs from collapsing by reducing surface tension. Surfactants may be derived from animal products, but synthetic surfactants with comparable effectiveness and potential cost advantages also exist [60]. Recent advances in surfactant treatments have focused primarily on ways to administer exogenous surfactants through minimally invasive or even noninvasive routes [60]. These include aerosolized delivery (nebulizer), laryngeal mask airway delivery, or delivery through a thin endotracheal catheter.

The 2019 European Consensus Guidelines recommend porcine over bovine surfactants and report that a dose of 200 mg/kg has greater therapeutic efficacy than 100 mg/kg [61]. The challenge with surfactant use in neonates is their safe administration, in particular, ways to obviate the need for intubation and bolus delivery [62]. Aerosolization of the agent appears promising, although the development of the appropriate equipment to nebulize molecules small enough to pass through the main neonatal airways and reach the alveoli has been an obstacle [62]. A current method allows sedation of the infant, intubation for surfactant administration, followed by rapid extubation [63].

The less-invasive surfactant administration (LISA) method inserts a small-size catheter through the vocal cords and into the infant’s trachea through which the surfactant is administered, whereupon the catheter is withdrawn [64]. LISA may be used to help spontaneously breathing preterm infants in order to reduce the need for mechanical ventilation. LISA is typically administered with noninvasive forms of respiratory support, such as CPAP and noninvasive positive-pressure ventilation (NIPPV) [65].

In an observational study (n=7,533) of very low birth weight neonates (gestational ages 22^0/7^ to 28^6/7^ weeks), one group never received surfactants (n=1214), one group was treated with LISA (n=2,624), and another group was administered surfactants by way of endotracheal tubes (n=3,695) [66]. The use of LISA was associated with significantly lower rates of mortality, BPD, intracerebral hemorrhage, and retinopathy of prematurity than the other groups. However, in infants under 26 weeks, there was an increased rate of focal intestinal perforation with LISA [66]. The advantage of LISA over other surfactant administration protocols is that it avoids the sedation and intubation of preterm infants. However, there is a risk that surfactant may be unequally distributed, for example, favoring one lung over the other, because of the difficulty in estimating the depth of catheter introduction into the trachea [62].

Nasal continuous positive airway pressure and nasal intermittent positive pressure ventilation

CPAP at 4-6 cmH_2_O has been demonstrated to be effective for obstructive but not central AOP [67]. NIPPV may be effective in treating AOP and preventing extubating failure [68]. There are no placebo-controlled studies of NIPPV for AOP infants, but NIPPV may avoid mechanical ventilation in infants with AOP [69]. In a study of 79 preterm infants randomized to NIPPV or NCPAP, the two groups had similar failure rates at 48 hours (13.5% vs. 15%, respectively) with a trend toward less surfactant use in the NIPPV group (32.4% vs. 53.7%, respectively) [70].

In a study of infants who were mechanically ventilated, 132 were randomized to receive biphasic nasal CPAP or regular nasal CPAP for 48 hours post-extubation [71]. The biphasic group had significantly fewer AOP events over 48 hours (p=0.002) and a higher rate of extubation success (p=0.074) with adverse events being similar between groups [71]. In a study of 19 preterm infants with AOP (mean gestational age of 30 weeks), neonates received flow-synchronized NIPPV, conventional NIPPV, or NCPAP for four-hour treatment sessions and then were crossed over [72]. All modes used a conventional nasal ventilator and synchronized flow with a pneumotachograph. The median event rate per hour was 2.9 (flow-synchronized NIPPV), 6.1 (regular NIPPV), and 5.9 (NCPAP), and central apneas per hour were 2.4, 6.3, and 5.4, respectively. Flow-synchronized NIPPV was more effective than conventional NIPPV or NCPAP in reducing desaturation, bradycardia, and central apnea episodes [72]. In another study, nasal CPAP was associated with a higher rate of failure than NPIVV in a study of 80 preterm infants [73].

Posture and positioning

Placing the neonate in the prone position has been shown to reduce AOP [74] and may also improve sleep in very premature infants [75]. Since prone sleeping also increases the risk of sudden infant death syndrome, care is to be taken that preterm infants sleep prone while in hospital but sleep supine after discharge [76]. A meta-analysis of five studies including 114 preterm infants treated by body positioning for AOP found no evidence that these body positioning changes decreased apnea or improved oxygen saturation [77].

Sensory stimulation

One of the oldest methods of treating AOP is still in common use: sensory stimulation. Sensory stimulation relies on tactile, auditory, and olfactory stimuli to help prevent or reduce apnea [78]. It is thought that the stimulation leads to excitatory neuronal activity in the brainstem, which, in turn, triggers respiratory drive [78]. The drawback to sensory stimulation is that it can arouse a neonate from sleep or disrupt sleep. In a study of olfactory stimulation, vanilla scent reduced AOP by 36% in 12/14 preterm infants with no side effects, but this study was conducted only over 24 hours and longer-term results are not known [79]. Kinesthetic stimulation, such as the use of an oscillating mattress, was not found to be effective in reducing AOP [80].

Stochastic resonance effects

Stochastic resonance therapy involves using noise to alter a biological system [81]. It had long been speculated that stochastic vibro-tactile stimulation may help regulate breathing patterns in preterm infants [82]. In a crossover study, special mattresses were equipped to offer preterm neonates with AOP mechanosensory stimulation by means of subarousal-level vibrations [83]. A total of 36 infants (mean gestational age of 30.5 weeks) were evaluated, and stochastic resonance treatment reduced apneic events by 50%, decreased oxygen desaturation, and reduced the intensity of bradycardic events by almost 20%. The procedure was noninvasive and well tolerated [83].

Head-to-head studies

Head-to-head studies of different treatment modalities or dosing regimens can be helpful in guiding clinical practice, but such studies are not frequent for AOP. A study comparing theophylline versus an oscillating waterbed for treating AOP was conducted in 20 preterm infants and found no significant differences between groups [84]. A meta-analysis of five clinical trials (n=108 total) comparing theophylline to caffeine for treating AOP in preterm infants found no differences between groups in terms of early treatment failure (one to three days), or later treatment failure (five to seven days) or the rate of apnea, but the caffeine groups had fewer adverse events [85]. In one study evaluating theophylline compared to mask CPAP for treating AOP, results were measured as a 50% reduction in apnea or the need to use an alternative treatment. The study found that for every 2.4 infants treated with mask CPAP instead of theophylline, there was one treatment failure. Thus, theophylline was more effective than mask CPAP for treating preterm infants with AOP [86].

A randomized study of 240 preterm infants (≤34 weeks) with AOP grouped neonates into those who received caffeine citrate (loading dose of 20 mg/kg followed by a daily maintenance dose of 5 mg/kg) and those who received aminophylline (loading dose of 5 mg/kg with a maintenance dose of 1.5 mg/kg every 8 hours). The aminophylline group had significantly fewer apneic spells after four to seven days of treatment (p=0.03), but the apnea rate and isolated desaturations were similar in days 1-3 of treatment, days 4-7, and days 8-14. Stays in the NICU were similar in both groups, and the mean heart rate was significantly faster in the aminophylline group (p<0.001) and aminophylline patients were at a greater risk for tachycardia. Thus, it appears that aminophylline is as effective as caffeine to prevent apneic spells in preterm infants with AOP, but aminophylline may have effects on the heart rate [87]. Compared to theophylline (n=100), caffeine was significantly more effective at reducing apnea events than theophylline over 21 days [88]. Results were similar when caffeine treatment was compared to aminophylline (n=67) [89]. In a meta-analysis of studies using theophylline (n=3 studies) and caffeine (n=3 studies) for AOP, these substances were effective in reducing the number of apneic events and reduced the need and time for mechanical ventilation [90]. In a meta-analysis of four trials (n=91 total) of preterm infants treated with either IV doxapram or methylxanthine over 48 hours, results were similar between groups and no adverse events were reported [91].

Preterm births are associated with higher morbidity and mortality rates compared to full-term births, but earlier gestational ages are seen in clinical practice. Since the heritability of AOP is 87% among same-sex twins, it is likely that AOP has a strong genetic component, which has not been entirely elucidated [92]. While AOP is often associated with an immature respiratory system, other factors such as infection, encephalopathy, intracranial hemorrhage, metabolic imbalances, or even body temperature may also be involved [2,93,94]. For many preterm infants, AOP resolves as the pulmonary system matures, but in other babies, apnea may persist into childhood [93].

Future directions in the care of AOP likely mean more studies to provide better and more specific guidance, but pharmacological innovation may be an important aspect. New agnostic respiratory stimulants in development may stimulate respiratory drive regardless of the etiology of the apnea. Such drugs are being considered for a variety of applications, including rescue from drug-induced respiratory distress, viral respiratory distress, and altitude sickness. The preterm population poses special challenges as this is an immature and highly vulnerable population at a particularly crucial stage of neurodevelopment. The role of AOP as a contributor to preterm infant morbidity and mortality has yet to be elucidated; however, it is not appropriate to relegate it to a benign condition simply because it tends to resolve as preterm infants mature. Interventions such as caffeine citrate may be effective but can have adverse effects and have been implicated in causing long-term damage to babies, while more conservative interventions such as prone posture, kangaroo care, and sensory stimulation may not be adequately effective. There is an unmet need for a treatment that is both effective and safe for this particular special population.

## Conclusions

Premature delivery is associated with increased morbidity and mortality. Many preterm infants have AOP, which tends to resolve as the neonate matures. Nevertheless, AOP may contribute to neonatal morbidity. A variety of treatment modalities exist with limited expert consensus to guide care. Pharmacological treatments, such as caffeine citrate and doxapram, are known to be effective but may be associated with side effects. The long-term effects of drug therapy in preterm infants are only beginning to be studied. Nonpharmacological treatments are often more conservative, safe, but less effective. Guidelines for therapeutic interventions in this population are scarce and even caffeine - a frequently used treatment - has no clear guidance in terms of dosing, when to start therapy, and when to discontinue it. New safe, effective, and appropriate treatments are needed for this vulnerable population.
